# Media Richness and Continuance Intention to Online Learning Platforms: The Mediating Role of Social Presence and the Moderating Role of Need for Cognition

**DOI:** 10.3389/fpsyg.2022.950501

**Published:** 2022-07-12

**Authors:** Zhen Wang

**Affiliations:** School of Foreign Languages, Zhejiang Gongshang University, Hangzhou, China

**Keywords:** media richness, continuance intention, social presence, online learning platforms, need for cognition

## Abstract

Continuance intention to online learning platforms has received increased attention in recent years, and media richness has been found to be an important antecedent influencing user retention. However, there is insufficient research on the mediating and moderating mechanism underlying this relation. The purpose of this article is to investigate (a) the positive association between three dimensions of media richness and user continuance intention, (b) the mediating role of social presence in the relationship between three dimensions of media richness and continuance intention, and (c) the moderating role of need for cognition in the relationship between three dimensions of media richness and social presence. We conduct a survey questionnaire with a sample of 368 users from online learning platforms and use structural equation modeling. The results indicate that the model proposed has a high explanatory power of continuance intention to online learning platforms and give support to the moderating role of the need for cognition. The study highlights the crucial role these technological-environment-related variables (media richness) have in relation to virtual user experience (social presence) and their continuance intention. Furthermore, this study shows the significance of differentiated online course design to foster users’ social presence according to user types.

## Introduction

Compared with conventional learning systems, online learning platforms have advantages in accessibility, permanency, immediacy, and interactivity, which can provide users with a convenient learning experience and satisfy their demands for high-quality educational resources. Therefore, more and more learners choose those platforms to continue their education. At present, online learning has a high commercial value and more and more institutions and training enterprises are providing online learning platforms. Thus, learners are facing an increasing number of online learning platforms, and the market is highly competitive. The managers of online platform focus on attracting learners to participate in the platform and reduce their attrition rates.

Previous researchers have examined the antecedents to users’ continuance intention within online learning platforms from the perspective of media richness and social presence. In terms of media richness, online learning platforms have provided information that is rich, reliable, and expressed in diversified ways. Hone and El Said (2016) suggested that rich massive open online course (MOOC) content was a significant predictor of MOOC retention. Arslan et al. (2022) posited that the more virtual professional learning network (VPLN) offered immediacy, personalization, and diversified support of learning activities, the more duration of learners’ in VPLN based on Twitter. In terms of social presence, online learning platforms have provided many rich technical methods through which users can communicate with other learners and instructors, such as real-time audio and video, augmented reality, and virtual reality. Hayashi et al. (2020) posited that social presence could better improve trainees’ continuance intention in e-learning systems. In the context of online learning platforms, the above-mentioned studies show media richness and social presence can influence users’ continuance intention. Furthermore, scholars have found that media richness effectively enhances users’ perception of social presence. Kim et al. (2016) indicated media richness of online learning platforms, especially the real-time communication tools, helps foster social presence. All those findings justify our research framework to include media richness and social presence to verify their influence on users’ continuance intention. Therefore, we suggest that social presence, which can be regarded as a learning experience for users (Cobb, 2009; Apostolellis and Bowman, 2016; Guo et al., 2021), mediates the effect of the technology environment (including the richness of media) on the continuous use of online learning platforms.

The need for cognition (NFC) is a psychological construct that concerns an individual’s tendency and enjoyment in engaging in effortful cognitive activity (Cacioppo and Petty, 1982). Zhang and Buda (1999) pointed out that, in some way, students’ cognitive efforts in solving difficult questions depend on their NFC. Some educational researchers have used NFC in relation to learning systems (Evans et al., 2003; Turner and Croucher, 2014; Cheong et al., 2022) since they are interested in how students learn and process information. However, to our knowledge, no previous research has analyzed the moderating role of NFC in online learning platforms. Given that these platforms provide abundant information for users to process and create a convenient social environment to facilitate information exchange and interact with each other, we consider that NFC is a relevant variable to understanding how media richness works in an online learning context.

This research is the first to explore the theory of media richness and social presence for the most commonly used online English learning platforms among Chinese college students. We specifically investigate how social presence mediates continuance intention with the media richness of online learning platforms. We discuss how media richness enhances users’ social presence, given that users are different in NFC. This article contributes to the literature on online education and education psychology by deepening our understanding of the relationship between online learning platforms and user behavior. Meanwhile, this study provides insights that are useful for managers of online learning platforms, as it advises how to optimize users’ online learning experience and enhance their continuance intention through media richness.

## Literature Review and Theoretical Background

### Media Richness

Daft and Lengel (1986) introduced media richness theory, defined as “the capacity of the medium to develop a common and shared meaning between the broadcaster and the receiver of the message.” Daft et al. (1987) then provide a theoretical framework to measure a certain communication media by its capacity to transmit rich information. By transmitting high-quality and diversified information, rich communication media can facilitate mutual communication and information exchanges between information sender and receiver. By adopting the definition proposed by Daft et al. (1987) and based on their criteria, Chen et al. (2004) suggest media richness of virtual stores can be measured by the following five criteria: (a) feedback to customers’ requests for product information, (b) different channels to present product information, (c) ability to clarify ambiguous issues about the product, (d) extent to which product comparison is allowed, and (e) consumer’s overall attitude about the information richness. Besides, Volery and Lord’s (2000) research further expands upon the necessity of rich media in online learning environments to provide effective content delivery. Based on the analysis of previous related research, this article adopts the definition of media richness proposed by Daft et al. (1987). We divide media richness in the context of online learning platforms into three dimensions: information expression richness, information content richness, and information quality richness. Information expression richness refers to the degree to which the information expression provided by the platform (such as text, pictures, and audio) meets the needs of learners. Information content richness measures the degree which extent the richness of the information content provided by the platform can meet the needs of learners. Information quality richness refers to the quality and reliability of the information provided by the platform to learners.

### Social Presence

Social presence was first conceptualized by Short et al. (1976) and was defined as “the salience of the interactants and their interpersonal relationship during a mediated conversation.” They argued that social presence is technologically determined, and different types of media had different forms of social presence. While Walther (1992) has rejected this technology-driven conceptualization of social presence and proposed that social presence is users’ feeling that other people are interacting with them while they are using the media. Based on this understanding, Rogers and Lea (2005) defined social presence as an individual’s perception of others’ warmth, social contact, familiarity, and intimacy, which arises from a sense of belonging and social recognition generated by their community interaction. In the online learning platform context, social presence can be regarded as users’ learning experience. We believe this experience is users’ own perception of a sense of belonging in a virtual learning community, which has been established during their discussing with or reacting to other learners or instructors *via* communication tools provided by platforms, such as real-time broadcast, screen sharing, and face-to-face chatting through audio and video.

### Continuance Intention

Continuance intention has an influential role in evaluating the success of information systems (Parthasarathy and Bhattacherjee, 1998; Bhattacherjee, 2001). Previous researchers have examined the antecedents to users’ continuance intention within online learning platforms from two perspectives. Some scholars argue that user perspective has a positive impact on online learning continuous intention, such as users’ personal characteristics (Roca et al., 2006; Safsouf et al., 2018) and users’ learning experiences (Zhao et al., 2020). While some scholars believe platform perspective can enhance users’ continuance intention, such as design and technique features of the platform (Yang et al., 2017; Chen et al., 2019) and content features of the platform (Hew and Cheung, 2014; Luo et al., 2017). However, few researchers have explored theoretical and empirical research on users’ continuance intention from the perspective of media richness and social presence, which are determined by the platform’s technological features.

## Research Hypotheses

### The Influence of Media Richness on Continuance Intention

Jo Black et al. (2002) argued that communication channel provided by financial services could be divided into high, medium, and low according to its ability to carry feedback, multiple cues, etc., and the high media richness rating one certain channel was, the more consumers would stick to it. Lan and Sie (2010) assessed a ubiquitous learning system and analyzed communication media richness with RSS, SMS, and email, their research found that the richer system’s information expression was, which in turn enhances learners’ communication, the more learners’ continuance intention toward a ubiquitous learning system. Zhu and Shen (2015) stated that managers of excellent network courses should pay attention to the novelty of courses’ information expression and ensure reliable courses information content; therefore, the majority of learners could satisfy their learning needs and finish those network courses. Gyamfi’s (2018) study concluded that there was a strong relationship between the breadth and depth of information provided by social media app and its usage for organizational learning. Chen et al. (2004) and Maity et al. (2018) argued that the reliability of product information provided by online shopping platforms could influence users’ understanding and trustworthiness of the product, and ultimately affect users’ continuance intention to this platform. Similarly, Cole and Timmerman (2015) pointed out learners would drop out of one MOOC platform as soon as they believed the reliability of learning resources provided by the platform was in doubt. Online platform builders with diversified notions will adopt specific technology to construct a platform, which leads to the differentiation in information expression, information content, and information quality of the platform. Therefore, those platforms vary in media richness as communication media, thus bringing different user experiences. When users participate in online learning or purchase something through online shopping, the user experience of different platforms cannot only affect their selection for this communication media but also influence their determination to whether or not to stay on the platform. Similar to the online financial service platform, ubiquitous learning system, online shopping platform, etc., the media richness of online learning platforms will also have a positive impact on users’ continuance intention. Based on these insights, we propose the following hypotheses.

H1a: Information expression richness has a positive effect on users’ continuance intention.

H1b: Information content richness has a positive effect on users’ continuance intention.

H1c: Information quality richness has a positive effect on users’ continuance intention.

### Social Presence Mediating Effect

Hassanein and Head (2007) found that socially rich text and pictures increased perceptions of social presence with online shopping. Aljukhadar et al. (2010) argued that the e-store’s information and media richness affected the perceived social presence. Allen et al. (2004) and Allen et al. (2013) proposed that media that are richer, might also more easily convey a sense of proximity and therefore be perceived as enabling more social presence when they investigated the impact of websites’ social presence on recruitment outcomes. Similarly, Carpentier et al. (2017) argued that the richer a hospital’s Facebook or LinkedIn page was, the more perceived social presence of nurses visiting the social media platform. Previous scholars mainly conducted research on media richness’s influence on social presence in the marketing context, but only a few studies on this topic have been carried out in the educational context. Oregon et al. (2018) argued that there was a distinct correlation between using rich media technologies and enhancing social presence in an online graduate program. Turk et al. (2022) posited students’ perceived teaching presence and social presence were enhanced *via* online learning environments compared to traditional offline classes.

Meanwhile, many related studies in different fields have verified the positive effect of social presence on users’ continuance intention. In the field of e-commerce, Li et al. (2003) found that when consumers perceived a high level of social presence in a virtual shopping website, they would have a better evaluation of a product, thereby showing a higher intention to choose the website. Lu et al. (2016) research also confirmed that the social presence of online social commerce marketplace has a positive impact on trusting beliefs, which in turn results in consumers’ stickiness to those marketplaces. Based on the peer-to-peer (P2P) accommodation platform, Ye et al. (2019) pointed out that social presence could enhance P2P customers’ willingness to continue the use of the platform. In the field of online information system, Song et al. (2008) and Choi et al. (2011) empirical research showed that social presence had a significant positive influence on online recommendation systems’ continuance intention. Besides, on some platforms of online education, previous scholars have confirmed the positive impact of social presence on continuance intention. Imlawi and Mafraq (2018) posited that online community design artifacts, that promote social presence in MOOCs websites, are critical to increasing online students’ participation intention. Song et al. (2019) suggested that by exploring social presence theory in course design and instruction on attrition in the graduate Recreation and Sport Administration online program at Western Kentucky University, the retention rates of undergraduate students were definitively higher than in prior programs. As the studies cited above show, we believe that the media richness of online learning platforms can be improved through various technical means. This can help learners establish friendly and intimate relationship with other learners or instructors in the online learning platform and stimulate more interactions. Psychologically, such relationships and interactions enhance learners’ sense of belonging within a virtual learning space and develop a feeling of being active members of a learning community, which in turn, drives learners to immerse in online learning. As time goes by, learners will choose to complete their learning on this platform. We thereby propose the following hypotheses.

H2a: Social presence mediates the relationship between information expression richness and continuance intention.

H2b: Social presence mediates the relationship between information content richness and continuance intention.

H2c: Social presence mediates the relationship between information quality richness and continuance intention.

### Need for Cognition Moderating Effect

Need for cognition (NFC) captures the extent to which individuals actively engaging in effortful reflection through seeking, evaluating, and integrating multiple relevant sources of information in arriving at an opinion (cognizers; individual with high NFC) or tend to form an opinion based on cursory or superficial aspects (cogmisers; individual with low NFC). Although NFC has not been shown to directly predict continuance intention, Cacioppo et al. (1996) suggested that NFC can influence an individual’s choice and acceptance of external information, and acceptance of the information’s depth and breadth. To avoid the greater cognitive effort required to process information, cognizers are more cline to ignore the content and quality of information and seek information sharing and discussion with others to accelerate their own comprehension. Previous studies have shown that in online shopping and online learning environments, users vary in NFC engaged in different information processing methods to form their own opinion. Liu and Shrum (2002) argued that customers with high NFC did not pay attention to the diversified information expression of advertising, so overemphasis on the interactive nature of advertising might not necessarily be beneficial to those customers to make a purchase decision. Kaynar and Amichai-Hamburger’s (2008) research showed that users with high NFC would pay more attention to the usefulness of network information than users with low NFC. Follow-up empirical research by Dickha̋user et al. (2009) showed that learners with low NFC tended to rely on external superficial clues, rather than the information itself they were receiving, to complete the information evaluation and analysis. During their information-processing stage, users varying in NFC will generate different user experiences toward the same dimension of media richness. Compared with other factors, such as collaborative learning strategy and communication skills, Del Barrio-Garciìa et al. (2015) pointed out that the richness of learning content provided by an e-learning environment can improve the perceived usefulness of students with high NFC. Chopik and Francis (2021) indicated that participants in high NFC were associated with a greater sense of ICT ownership when they were facing abundant ICT interactive interfaces. As the studies cited above show, compared with information expression richness, information content richness and information quality richness provided by the platform require users to devote more to engaging in cognitively challenging tasks and effortful thinking. Therefore, we believe that users with high NFC will pay more attention to information content richness and information quality richness. They are attracted by the abundant information provided by the platform at first sight, then satisfied with the reliability of those information. This kind of user cares more about a quick refresh of learning resources and fast verification of trustworthy information, which give them the feeling of being together with others in the same learning community. If they resorted to knowledge sharing, even the simplest text posts can effectively stimulate their social presence. The real-time communication tools are not appealing to them. While users with low NFC pay more attention to the information expression richness of the platform. Because they tend to avoid in-depth information processing, they prefer information expressed vividly and concisely and overlook the content and quality of information. When they must engage in cognitive activity to make their own decisions, turning to for help is their first choice. This kind of user relies heavily on interpersonal interaction with the support of various information expression methods, which improve their sense of social presence greatly. Based on these insights, we propose the following hypotheses.

H3a: Users with high NFC negatively moderate the relationship between information expression richness and social presence.

H3b: Users with high NFC positively moderate the relationship between information content richness and social presence.

H3c: Users with high NFC positively moderate the relationship between information quality richness and social presence.

**FIGURE 1:**
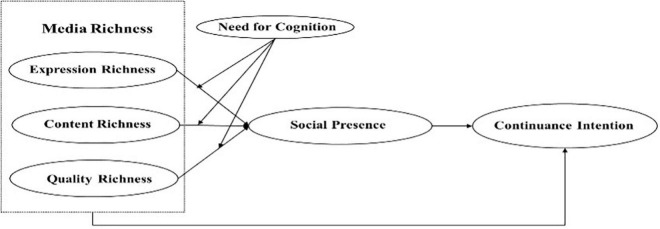
Conceptual model.

In Figure 1, we outline the conceptual model for this study.

## Materials and Methods

### Data Collection

Data for this study were collected through survey questionnaires. The questionnaire consisted of two parts, the background information of the interviewees and measurements of key variables. We analyzed the most commonly used online learning platforms used by college students in China, such as the Chinese MOOC platform^1^, Hujiang class^2^, and Tencent class^3^. To ensure sample users are from each of these platforms and obtain a sufficient sample size, we employed “WJX” paid questionnaire service. “WJX” is a professional online questionnaire survey company and has been widely used by scholars in China. The “WJX” platform conducted the online survey from April 12, 2019 to April 28, 2019, 675 questionnaires were sent out through QR code or WeChat Mini Program. A total of 328 valid questionnaires were obtained. The users in our sample were college students; the demographic information was shown in Table 1.

**TABLE 1:** Demographics.

Demographic	Range	Frequency	Percentage (%)
Age	20–25	287	87.51%
	26–30	8	2.44%
	31–35	13	3.96%
	36–40	11	3.35%
	41–45	9	2.74%
Gender	Male	109	33.23%
	Female	219	66.77%
Education level	Graduate level degree	45	13.72%
	Bachelor degree	283	86.28%
Total responses		328	100%

### Data Analysis

In this study, a seven-point Likert scale was used to measure key variables. We measured three dimensions of media richness, namely, information expression richness, information content richness, and information quality richness. These three dimensions were measured using the metrics developed by Vickery et al. (2004) and by Liu et al. (2009), each dimension consisting of three questioning items, respectively. With respect to social presence, five items were adopted from Heeter (1992) and Molinillo et al. (2018). The measurement of continuance intention was borrowed from Bhattacherjee (2001), which consists of four items. The measurement of the NFC was based on the methods used by Cacioppo et al. (1996), which consists of five items. These items are summarized in Table 2.

**TABLE 2:** Reliability and validity analysis.

Variables	Measurement Items	Loading	C.R.	*P*
Expression richness α = 0.816 CR = 0.818 AVE = 0.60	The platform provides information on a variety of expressions (text, pictures, audio and video, emoticons, etc.).	0.797	–	
	I can use a variety of expressions to participate in information sharing, interacting with others in the platform.	0.747	12.472	***
	The platform meets my requirements for information expression.	0.778	12.770	***
Content richness α = 0.847 CR = 0.856 AVE = 0.6665	The platform provides a wide range of information.	0.867	–	
	The platform provides in-depth information.	0.830	16.367	***
	The platform meets my requirements for information content.	0.744	14.612	***
Quality richness α = 0.859 CR = 0.843 AVE = 0.643	The platform provides high-quality information.	0.848	–	
	The platform provides trustworthy information.	0.810	15.812	***
	The information provided by the platform can help me.	0.801	15.641	***
Social presence α = 0.878 CR = 0.875 AVE = 0.669	When using the platform, I have a feeling of being with others.	0.814	–	
	When using the platform, I have a sense of sociality.	0.802	15.671	***
	When using the platform, I feel like chatting face-to-face with others.	0.837	16.458	***
	When using the platform, I feel very warm.	0.652	12.108	***
	When using the platform, I have a sense of belonging.	0.701	13.235	***
Continuance intention α = 0.867 CR = 0.868 AVE = 0.619	I intend to continue using the platform.	0.795	–	
	I will use the platform on a regular basis in the future.	0.798	14.904	***
	I will frequently use the platform in the future.	0.767	14.258	***
	I will strongly recommend others to use the platform.	0.792	14.775	***
Need for cognition α = 0.903 CR = 0.904 AVE = 0.677	I would prefer complex to simple problems.	0.789	–	
	Thinking is my idea of fun.	0.857	16.935	***
	I really enjoy a task that involves coming up with new solutions to problems.	0.821	16.083	***
	The notion of thinking abstractly is appealing to me.	0.737	14.069	***
	I would prefer a task that is intellectual, difficult, and important to one that is somewhat important but does not require much thought.	0.833	16.377	***

Fitting index: P = 0.000, χ^2^/df = 1.885, GFI = 0.905, PNFI = 0.768, IFI = 0.955, CFI = 0.954, RMSEA = 0.052. ***P < 0.001.

### Common Method Variance

In this study, Harman’s single-factor method was used to perform a common method variance bias test on the sample data. The factor analysis results show that 6 factors with eigenvalues greater than 1 were extracted without rotation, and the load of the first factor was 29.432%, which was lower than the acceptable standard of 40%. Therefore, common method variance is not a concern here.

## Results

### Reliability and Validity

Tables 2, 3 show the reliability and validity test results of our sample data. The Cronbach’s α coefficients of each variable are greater than 0.8, indicating the scale has good internal consistency. According to Chin, Chin and Salisbury (1997) and Hair et al. (2009), the standardized factor loading of each measurement item is greater than 0.6, the critical ratio value is greater than 1.96, the composite reliabilities values are greater than 0.8, and the average variances extracted AVE are greater than 0.5, thereby providing evidence of convergent validity. The square root of the AVEs in the diagonal line is greater than the correlation coefficients of the corresponding row and column, indicating good discriminate validity of our measurement model. The fitting indexes of the measurement model all meet the recommended requirements, showing that the model has good fitting.

**TABLE 3:** Square root of AVE and correlation coefficient matrix.

Variable	ER	CR	QR	SP	CI	NFC
ER	**0.775**					
CR	0.420	**0.815**				
QR	0.317	0.514	**0.820**			
SP	0.059	0.101	0.121	**0.765**		
CI	0.353	0.386	0.424	0.040	**0.788**	
NFC	0.404	0.461	0.462	0.124	0.421	**0.809**

*ER, expression richness; CR, content richness; QR, quality richness; SP, stands for social presence; CI, continuance intention; NFC, need for cognition. Bold values means the square root of the AVEs which shown in the diagonal line.*

### Hypothesis Testing

**FIGURE 2:**
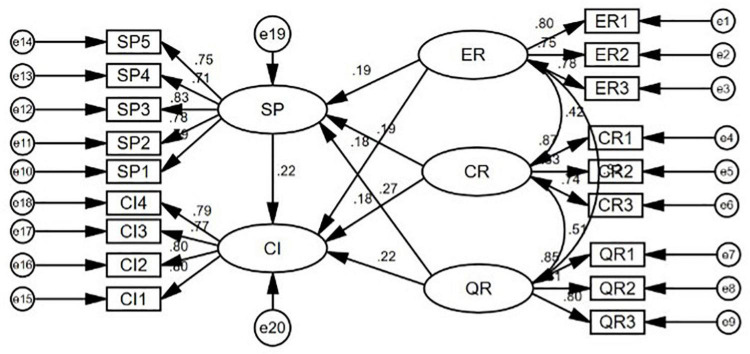
Structural equation model.

We use AMOS22.0 for the structural equation model analysis of the sample data in order to verify our research hypotheses. The analysis result of the structural equation model is shown in Figure 2 and in Table 4. The fitting index of the model shows that χ^2^/*df* is 2.404 < 3, RMSEA is 0.066 < 0.08, SRMR is 0.054 < 0.08, IFI(0.944), TLI(0.932), GFI(0.903), and CFI(0.943) are all greater than the reference value of 0.9. The corresponding critical ratio values of the standardized path coefficient among latent variables are greater than 1.96, which are of statistical significance at the *P* = 0.05 level at least. The results of the structural equation model confirm hypotheses H1a, H1b, and H1c.

**TABLE 4:** Analysis results of structural equation model.

Hypothesis path				Standardized path coefficient			C.R.	*P*
Social presence ← Expression richness				0.189			2.814	0.005
Social presence ← Content richness				0.195			2.634	0.008
Social presence ← Quality richness				0.272			3.835	***
Continuance intention ← Expression richness				0.179			2.720	0.007
Continuance intention ← Content richness				0.184			2.549	0.011
Continuance intention ← Quality richness				0.216			3.073	0.002
Continuance intention ← Social presence				0.221			3.373	***
**Fitting Index**	***P***	**χ2/df**	**RMSEA**	**IFI**	**SRMR**	**TLI**	**GFI**	**CFI**
	0.000	2.404	0.066	0.944	0.054	0.932	0.903	0.943

***Indicates a significance level P < 0.001.

To better illustrate the mediation effect of social presence, we use the bootstrap method to analyze the data (Shrout and Bolger, 2002; Preacher and Hayes, 2008). We set the number of bootstrap samples for percentile confidence intervals at 5,000 and the level of confidence at 95%. Table 5 indicates the results of mediation effect testing. The indirect effect of expression richness on continuance intention is 0.042 and the effect is significant as the interval excludes zero (0.008, 0.108), which shows social presence plays a significant role in the relationship between expression richness and continuance intention. The indirect effect of content richness on continuance intention is 0.043 and the effect is significant as the interval excludes zero (0.007, 0.118), which shows social presence plays a significant role in the relationship between content richness and continuance intention. The indirect effect of quality richness on continuance intention is 0.060 and the effect is significant as the interval excludes zero (0.019, 0.133), which shows social presence plays a significant role in the relationship between quality richness and continuance intention. Thus, hypotheses H2a, H2b, and H2c are confirmed.

**TABLE 5:** Analysis results of mediation effect.

Indirect path	Indirect effect	Boot LLCL	Boot ULCL
Expression richness → Social presence → Continuance intention	0.042	0.008	0.108
Content richness → Social presence → Continuance intention	0.043	0.007	0.118
Quality richness → Social presence → Continuance intention	0.060	0.019	0.133

We use PROCESS to test the moderator effect of the NFC through the Bootstrap method proposed by Preacher et al. (2007). We set the number of bootstrap samples for percentile confidence intervals at 5,000 and the level of confidence at 95%. We take expression richness as the independent variable, social presence as the dependent variable, and NFC as the moderating variable, while gender and age as the control variables. The result in Table 6 shows the model is significant (*R*^2^ = 0.2404, *P* = 0.000), and the moderator effect is also significant as the interval excludes zero (−0.1605, −0.0151). The moderator effect is −0.0878, which means high NFC negative moderates the relationship between expression richness and social presence, thus hypothesis H3a is confirmed. We take content richness as the independent variable, social presence as the dependent variable, and NFC as the moderating variable, while gender and age as the control variables. The result shows that the model is significant (*R*^2^ = 0.2415, *P* = 0.000), and the moderator effect is also significant as the interval excludes zero (0.0205, 0.1827). The moderator effect is 0.1016, which means high NFC positive moderates the relationship between content richness and social presence, thus hypothesis H3b is confirmed. We take quality richness as the independent variable, social presence as the dependent variable, and NFC as the moderating variable, while gender and age as the control variables. The result shows the model is significant (*R*^2^ = 0.2467, *P* = 0.000), and the moderator effect is also significant as the interval excludes zero (0.0325, 0.1707). The moderator effect is 0.1016, which means high NFC positive moderate the relationship between quality richness and social presence, thus hypothesis H3c is confirmed.

**TABLE 6:** The analysis results of the moderator effect.

Variable	Model 1	Model 2	Model 3
Gender	0.0468	0.0505	0.0157
Age	0.0190	−0.0079	−0.0049
Expression richness	0.5270**	0.1707**	0.1505**
Content richness	0.2728***	−0.1682	0.2536**
Quality richness	0.2437*	0.1986**	−0.2188
NFC	0.4208**	−0.4341*	−0.4485**
Expression richness × NFC	−0.0878*		
Content richness × NFC		0.1016*	
Quality richness × NFC			0.1016**
Bootstrap 95%	[−0.1605, −0.0151]	[0.0205, 0.1827]	[0.0325, 0.1707]
*R* ^2^	0.2404***	0.2415***	0.2467***
*F*	14.471***	14.5515***	14.9750***

***P < 0.001, **P < 0.01, *P < 0.05.

## Conclusion

### Discussion

Although the influence of media richness and social presence on users’ continuance intention toward online learning platforms has begun to gain empirical support (Hone and El Said, 2016; Hayashi et al., 2020), there is insufficient evidence concerning the underlying mediating and moderating mechanisms. This study empirically proposed and verified a research model to test whether media richness would directly associate with continuance intention, meanwhile indirectly associated with continuance intention through social presence. Besides, the research model tested whether users’ perception of social presence was moderated by the NFC. Our results confirmed all expected hypotheses and strongly supported the model we proposed.

Regarding media richness and social presence, the correlation analyses indicated that three dimensions of media richness positively predicted users’ continuance intention, which supports existing evidence of the critical role media richness plays in this regard (Cole and Timmerman, 2015; Zhu and Shen, 2015; Gyamfi, 2018). Mediation analyses revealed that social presence partially mediated the relation between three dimensions of media richness and continuance intention. That is, media richness would positively predict social presence, and in turn, social presence could positively predict continuance intention. As far as we know, this is the first study to report such results in the context of online learning.

Regarding the moderating role of users’ NFC, our results showed users with high NFC negatively moderate the relation between information expression richness and social presence, while positively moderate the relations between the other two dimensions of media richness and social presence. For high-NFC users, the commonly accepted diversified expression of learning resources cannot enhance their social experience toward the online learning community.

### Theoretical Implications

This study provides novel insights into the underlying mechanisms of how technological factors (media richness) influence continuance intention through mediating factors (social presence) related to virtual user experience. In addition, this study analyses the moderating role of NFC. The theoretical contributions of this article are as follows.

First, our research findings further extend previous media richness literature by examining the specific relations among the three dimensions of media richness and continuance intention toward online learning platforms. This finding supports the previous tentative research on the online learning context (e.g., Lan and Sie, 2010; Cole and Timmerman, 2015; Zhu and Shen, 2015). Furthermore, by dividing media richness into three dimensions and confirming their positive association with continuance intention, we have verified the validity of media richness theory with more recent communication channels. Since media richness theory was developed in the mid-1980s, advanced communication technology has been raising more challenges toward its application in concerning research field.

Second, this study also contributes to media richness and social presence literature by identifying social presence as a new mediator in the relation between media richness and continuance intention. This new mediator and its links to dimensions of media richness extend our knowledge that technical features of online learning platforms facilitate the development of media richness, and consequently stimulate users’ positive feelings toward interacting with instructors or other users, which enhance their sense of social presence, and ultimately affect users’ continuance intention. As previous research showed that current media easily allow virtual community’s establishment and communicators’ interactivity, which significantly facilitates users’ virtual experiences. Thus, our findings have the potential to extend the research area of fostering positive user experience toward online learning platforms *via* communication technology.

Third, we successfully explained the moderating role of users’ NFC, which supports the previous research on applying NFC in the education context (e.g., Zhang and Buda, 1999; Evans et al., 2003; Turner and Croucher, 2014; Del Barrio-Garciìa et al., 2015; Cheong et al., 2022). No previous research has proposed that NFC, as the impact of human’s intrinsic factors in accepting subjective user experience, could moderate the relation between media richness and social presence, therefore our findings enrich the extant psychology and consumer behavior literature by revealing intrinsic factors in explaining human reactions to information systems.

### Managerial Implications

The results of this study are important for online learning platform developers and instructors who dedicate to decrease users’ high attrition rates.

First, our findings suggest that platform developers should implement system features to enhance social presence and encourage course continuance. We recommend platform designers and course instructors to ensure a high level of three dimensions of media richness in their education information. From the expression richness perspective, the integration of traditional and advanced technical methods, such as combing texts, emoticons with virtual reality gear can satisfy users’ need to share learning experiences and exchange opinions. From the content richness perspective, the combination of the national public service platform for educational resources founded by the minister of education of China and learning information provided by institutions, educators, and user-generated content (UGC) can enlarge users’ searching scope. From the quality richness perspective, the inclusion of artificial intelligence technology can enable users to quickly certificate the trustworthiness of learning information. Meanwhile, all those implemented system features can also help users establish coordinated learning communities and facilitate interactions among users and instructors, which in turn, enhance users’ social presence. Therefore, besides the discussion forum, developers should incorporate social-affordance devices which enjoy great popularity in the mobile internet era, such as Twitter, Facebook, QQ, WeChat, and TikTok.

Second, our findings suggest developers should match different dimensions of media richness to the users’ characteristics. NFC can moderate users’ perception of social presence when they are facing the same dimension of media richness, which in turn, affect the continuance intention of an online learning platform. This highlights the need for managers to take actions to avoid undesired effects. Users’ NFC profiles could be established at the beginning of the online course to provide individualized interface displays, tool options, and services. For groups composed of high NFC users, it is advisable to offer guidelines, checklists, databases, or templates to provide sufficient and in-depth information, and it is needless to take any strenuous efforts indulging in vivid information displaying that full of animation. Managers should incorporate the links to learning materials that can track authoritative sources. *Via* those technical means, high NFC users will be attracted by the credible information. Meanwhile, the level of expression richness in platforms’ education resources and communication supports should vary from high NFC users to low NFC users. For the high NFC user group, managers can assign educators or instructors of seniority to reply to their queries or join their discussion, but compared to low NFC users, the immediate customer reply is not their priority. For groups composed of low NFC users, all they need is diversified information expression. Therefore, online learning platform developers should place a great emphasis on satisfying their needs. In addition to those above-mentioned traditional technical means, customer intelligence and artificial intelligence for customer interactions or group discussions should be embedded in platforms as soon as possible.

### Research Limitations and Future Prospects

Our study has some limitations. First, we conducted this research in a Chinese context only, therefore, the generalization of research findings to online learning platforms and participant profiles from different cultural and geographical backgrounds should be performed with caution. Further research should recruit large samples and conduct the survey in different countries to determine whether the findings of this study apply to different contexts. Second, our data sample mainly consists of college students using online learning platforms. This restricted sample affects the universal applicability of our research conclusions to a certain extent. Future research is needed to enlarge the scope of online learning platforms’ users. Third, for this time we did not conduct the moderated mediation analysis on our proposed model.

We suggest that future studies should estimate further the first-stage moderating effect of the NFC on the relationship between media richness and social presence and the second-stage moderating effect of the NFC on the relationship between social presence and continuance intention. Therefore, we can obtain a comprehensive understanding of continuance intention based on the remaining unexplained variance in our future research model.

## Data Availability Statement

The original contributions presented in this study are included in the article/supplementary material, further inquiries can be directed to the corresponding author.

## Author Contributions

ZW contributed to conception and design of the study, organized the database and performed the statistical analysis, wrote the first draft of the manuscript, contributed to the manuscript revision, read, and approved the submitted version.

## Conflict of Interest

The author declares that the research was conducted in the absence of any commercial or financial relationships that could be construed as a potential conflict of interest.

## Publisher’s Note

All claims expressed in this article are solely those of the authors and do not necessarily represent those of their affiliated organizations, or those of the publisher, the editors and the reviewers. Any product that may be evaluated in this article, or claim that may be made by its manufacturer, is not guaranteed or endorsed by the publisher.

## Footnotes

1
http://icourse163.org

2
http://class.hujiang.com

3
http://ke.qq.com
